# Increased sensitivity to chemically induced colitis in mice harboring a DNA-binding deficient aryl hydrocarbon receptor

**DOI:** 10.1093/toxsci/kfac132

**Published:** 2022-12-15

**Authors:** Karoline Alvik, Peng Shao, David Hutin, Carolyn Baglole, Denis M Grant, Jason Matthews

**Affiliations:** Department of Nutrition, Institute of Basic Medical Sciences, University of Oslo, Oslo, Norway; Department of Pharmacology and Toxicology, University of Toronto, Toronto M5S1A8, Canada; Department of Pharmacology and Toxicology, University of Toronto, Toronto M5S1A8, Canada; Department of Medicine, McGill University, Montreal H4A3J1, Canada; Department of Pathology, McGill University, Montreal H4A3J1, Canada; Department of Pharmacology and Therapeutics, McGill University, Montreal H3G1Y6, Canada; Department of Pharmacology and Toxicology, University of Toronto, Toronto M5S1A8, Canada; Department of Nutrition, Institute of Basic Medical Sciences, University of Oslo, Oslo, Norway; Department of Pharmacology and Toxicology, University of Toronto, Toronto M5S1A8, Canada

**Keywords:** aryl hydrocarbon receptor, DNA-binding domain, dextran sulfate sodium, gut immunity, microbiota, AHR

## Abstract

The aryl hydrocarbon receptor (AHR), a transcription factor best known for mediating toxic responses of environmental pollutants, also integrates metabolic signals to promote anti-inflammatory responses, intestinal homeostasis, and maintain barrier integrity. AHR regulates its target genes through direct DNA-binding to aryl hydrocarbon response elements (AHREs) but also through tethering to other transcription factors in a DNA-binding independent manner. However, it is not known if AHR’s anti-inflammatory role in the gut requires its ability to bind to AHREs. To test this, we determined the sensitivity of *Ahr^dbd/dbd^* mice, a genetically modified mouse line that express an AHR protein incapable of binding to AHREs, to dextran sulfate sodium (DSS)-induced colitis. *Ahr^dbd/dbd^* mice exhibited more severe symptoms of intestinal inflammation than *Ahr^+/+^* mice. None of the *Ahr^dbd/dbd^* mice survived after the 5-day DSS followed by 7-day washout period. By day 6, the *Ahr^dbd/dbd^* mice had severe body weight loss, shortening of the colon, higher disease index scores, enlarged spleens, and increased expression of several inflammation genes, including interleukin 1b (*Il-1b*), *Il-6*, *Il-17*, C-x-c motif chemokine ligand 1 (*Cxcl1*), *Cxcl2*, Prostaglandin-endoperoxide synthase (*Ptgs2*), and lipocalin-2. Our findings show that AHR’s DNA-binding domain and ability to bind to AHREs are required to reduce inflammation, maintain a healthy intestinal environment, and protect against DSS-induced colitis.

The aryl hydrocarbon receptor (AHR) is a ligand-dependent transcription factor and member of the basic helix-loop-helix (bHLH)-per-ARNT-sim (PAS) family (Gu *et al.*, 2000; Hankinson, 1995). AHR is best known for its role in 2,3,7,8-tetrachlorodibenzo-*p*-dioxin (TCDD) toxicity but has emerged as an important factor that transmits environmental and endogenous signals to dampen inflammation and regulate immune cell homeostasis (Stockinger *et al.*, 2014; 2021). Several endogenous and dietary ligands or activators of AHR have been identified, including the tryptophan metabolites kynurenine and 6-formylindolo(3,2-b)carbazole (FICZ), as well as dietary ligands such as indole-3-carbinol and one of its acid condensation products, 3,3′-diindolylmethane (Avilla *et al.*, 2020; DeGroot *et al.*, 2015; Denison and Nagy, 2003; Seok *et al.*, 2018). In the canonical AHR pathway, ligand binding to AHR causes its translocation to the nucleus where it heterodimerizes with AHR nuclear translocator (ARNT). The AHR-ARNT complex binds to aryl hydrocarbon response elements (AHREs; xenobiotic or dioxin response elements) in the regulatory regions of its target genes, including the drug-metabolizing enzymes cytochrome P450 1A1 (*CYP1A1*) and *CYP1B1*, cytokines, growth factors, and cell cycle regulators (Hankinson, 2005).

AHR is associated with several inflammatory and immune disorders, including inflammatory bowel disease (IBD), allergic responses, cardiovascular disease, multiple sclerosis, and rheumatoid arthritis (Hui and Dai, 2020; Sartor, 2006; Wheeler *et al.*, 2017; Yi *et al.*, 2018). Because of this, there is considerable interest in targeting AHR to constrain inflammation (Cannon *et al.*, 2021; Pernomian *et al.*, 2020). For example, tapinarof, an AHR agonist, has recently been approved for the treatment of plaque psoriasis and atopic dermatitis (Keam, 2022).

AHR is associated with ulcerative colitis and Crohn’s disease, which are the 2 major forms of IBD. Immune cells isolated from patients suffering from Crohn’s disease have reduced levels of AHR (Monteleone *et al.*, 2011). Treatment with the natural AHR ligand, *Indigo Naturalis*, for 8 weeks resulted in effective clinical responses in patients with ulcerative colitis (Naganuma *et al.*, 2018). In mouse models of intestinal inflammation, AHR activation significantly improves, while its loss or the reduction of its endogenous ligand levels exacerbates dextran sulfate sodium (DSS)-induced colitis and bacterial-induced mucosal inflammation (Lamas *et al.*, 2016; Li *et al.*, 2011; Monteleone *et al.*, 2011; Schiering *et al.*, 2017). In addition, commensal microbial products such as indole-3-pyruvic acid, urolithin A, short-chain fatty acids, and dihydroxyquinoline may regulate intestinal inflammation in an AHR-dependent manner (Pernomian *et al.*, 2020).

Previous studies have reported that AHR regulates some cellular pathways, and the expression of inflammatory genes, through tethering to other transcription factors in an AHRE-independent manner (Beischlag *et al.*, 2008; Dere *et al.*, 2011). This has led to several proposed models in which AHR regulates cellular events through noncanonical signaling processes that are independent of nuclear translocation, AHRE binding or ARNT dimerization (Grosskopf *et al.*, 2021; Puga *et al.*, 1997; Wright *et al.*, 2017; Zhu *et al.*, 2018). Genome-wide AHR ChIP-chip and Chip-sequencing analyses revealed that approximately 50% of AHR-enriched regions do not contain an AHRE (Dere *et al.*, 2011; Lo and Matthews, 2012). These results further support the notion that tethering to other transcription factors is an important function of AHR regulation in addition to its direct binding to AHREs. Genetically modified mice that express a mutant AHR that is incapable of binding to AHREs, termed *Ahr^dbd/dbd^* mice, are resistant to TCDD toxicity and exhibit the same ductus venous development abnormalities that have been reported for *Ahr^−/−^* mice (Bunger *et al.*, 2008). These findings confirm the importance of AHR-AHRE binding in developmental and toxicological AHR signaling. However, it is yet to be determined if AHR’s anti-inflammatory role in the gut is dependent on its ability to bind to AHREs.

Here, we used a DSS-induced colitis model to investigate the effect of loss of AHR’s DNA-binding activity on DSS-induced intestinal inflammation. We observed that DSS-exposed *Ahr^dbd/dbd^* mice exhibited increased severity of disease symptoms. Our findings provide the first evidence that DNA-binding deficient AHR increases intestinal inflammation in the DSS model of colitis. These data further support the importance of the canonical AHR pathway in the biological and toxicological signaling of AHR.

## Materials and methods

###  

####  

##### Chemicals

DSS salt reagent grade (MP Biomedicals) was dissolved to a concentration of 2% in autoclaved drinking water. TCDD was purchased from Accustandard (New Haven, Connecticut). Dimethyl sulfoxide (DMSO) and all other chemicals and biological reagents were purchased from Merck (Frankfurt, Germany) unless stated otherwise.

##### Animals, TCDD, and DSS treatment

For all studies, 8-week-old male *Ahr^+/+^* and *Ahr^dbd/dbd^* mice were used, which were a generous gift from Christopher Bradfield, McArdle Laboratory for Cancer Research University of Wisconsin, Madison, Wisconsin. The characterization and generation of the *Ahr^dbd/dbd^* mice have been described previously (Bunger *et al.*, 2008). The *Ahr^+/dbd^* heterozygotes were bred together to give *Ahr^dbd/dbd^* and *Ahr^+/+^* littermates that were used for experiments. Tissues for genotyping were clipped from tails at 2 weeks of age. DNA extraction and PCR reactions were performed using the REDExtract-N-Amp tissue PCR kit using forward 5′-CTGAGGGGACGTTTTAATG-3′ and reverse 5’-AACATTTGCACTCATGGATAG-3′ primers and following the manufacturer’s recommendations. The PCR amplicon was digested with *Bam*HI, because a *Bam*HI recognition sequence was introduced in the *Ahr^dbd/dbd^* sequence. The digestion reaction was then separated on a 2% agarose gel. For the TCDD treatment studies, male *Ahr^+/+^* and *Ahr^dbd/dbd^* littermates were intraperitoneally injected with 100 µg/kg TCDD or an equivalent volume of DMSO vehicle control. After 2, 6, or 24 h, animals were sacrificed by cervical dislocation. Liver tissues were flash frozen in liquid nitrogen immediately after collection and later stored at −80°C. For the DSS studies, male *Ahr^+/+^* and *Ahr^dbd/dbd^* mice were housed singly and given either normal drinking water or water containing 2% (w/v) DSS for 5 days to induce IBD (Mizoguchi, 2012). The DSS-containing water was replaced with regular water after 6 days and the mice were monitored until day 12. Water and food consumption were measured daily. Body weight and signs of IBD were monitored daily, including diarrhea, weight loss, dehydration, hematochezia, weakness, and rectal prolapse. The animals received supportive care if needed, which included, but was not limited to a heating pad, mash and/or other fluid supplementation. At the end of the experiment animals were euthanized, and liver, intestines, and colon were removed for gene expression, histological, and biochemical analyses. Feces were collected immediately before the start of the experiment and used to evaluate changes in the microbiome by a real-time qPCR-based method, as we and others have described (Hutin *et al.*, 2022; Lamas *et al.*, 2016; Yang *et al.*, 2015). All animals were bred and cared for at the University of Toronto. Care and treatment of animals followed the guidelines set by the Canadian Council on Animal Care and were approved by the University of Toronto Animal Care Committee.

##### Ahr gene cloning and sequencing

The liver RNA of one *Ahr^dbd/dbd^* mouse and one *Ahr^+/+^* mouse was extracted using Aurum total RNA mini kit, and cDNA was synthesized using Applied Biosystems high-capacity cDNA reverse transcription kit according to the manufacturer’s instructions. An *Ahr* gene-specific reverse primer with an *Xho*I restriction site (5′-AATTCTCGAGCTACAGGAATCCACCAGG TGTGATATC-3′) was used instead of the 10× RT random primers. *Ahr* cDNA was then PCR amplified using Platinum *Pfx* DNA polymerase using the forward primer containing a *Nhe*I site: 5′-AATTGCTAGCGCCACCATGAGCAGCGGCGCCAACATCAC-3′, and the *Ahr* gene-specific reverse primer. The *Ahr* PCR products and the pcDNA3.1 plasmids were then cloned into the *Nhe*I and *Xho*I sites of pcDNA3.1. The complete *Ahr* mRNA sequence from *Ahr^+/+^* and *Ahr^dbd/dbd^* was determined by the Centre for Applied Genomics (TCAG) operated by the Hospital for Sick Children (SickKids) (Toronto, Ontario, Canada).

##### Luciferase reporter gene assay

COS-1 cells were plated on 12-well dishes at a density of 1.00 × 10^5^ to 1.25 × 10^5^ cells with 1 ml media per well. The following day, each well was transfected with 2 µl Lipofectamine 2000 and 1 µg DNA consisting of 300 ng pGudLuc 4.1, 100 ng pCH110, and 50 ng pEGFP, 400 ng of pcDNA3.1-Ahr^wt^ or pcDNA3.1-Ahr^dbd^. pGudLuc 4.1 is an AHR-driven reporter construct containing a luciferase gene downstream of the *Cyp1a1* promoter. After 6 h, every well was treated with either DMSO or 100 nM TCDD. The next morning, the cells were lysed, and the luciferase activity was measured using ONE-Glo luciferase assay and normalized to β-galactosidase activity. To confirm the expression of AHR^wt^ and AHR^dbd^ in COS-1 cells after transfection, COS-1 cells were again plated on 12-well dishes at a density of 1.00 × 10^5^ to 1.25 × 10^5^ cells with 1 ml medium per well. On each plate, 4 wells were transfected with each of pcDNA3.1, pcDNA3.1-Ahr^dbd^, and pcDNA3.1-Ahr^wt^. The cells were washed with PBS once, and then scraped and suspended in 250 ml RIPA buffer as described below.

##### RNA extraction, cDNA synthesis, and RT-qPCR

The Aurum total RNA mini kit was used for RNA extraction (BioRad, Hercules, California). For the liver tissue, 20–40 mg liver tissue was homogenized in 700 µl lysis solution. For colon tissue, fecal matter was pushed out, and the tissue was flushed with PBS. The samples were divided in 3, flash frozen in liquid nitrogen and then stored at −80°C. The distal end of the colon was homogenized in 500 µl TRizol and after extraction with 100 µl chloroform and centrifugation, the upper layer was transferred to a new tube containing 300 µl of 70% ethanol. The RNA was added to Aurum RNA purification columns and the RNA was purified according to the manufacturer’s instructions. One milligram of total RNA was reverse transcribed with High-capacity cDNA reverse transcription kit using standard conditions (Applied Biosystems). For real-time qPCR, 1 µl of cDNA was amplified with 5 µl KAPA SYBR fast qPCR master mix (2×) universal, 0.1 µl forward primer at 10 µM, 0.1 µl reverse primer at 10 µM, and 3.8 µl water. The cycling conditions were 95°C for 3 min, followed by 45 cycles of 95°C for 10 s and 60°C for 20 s. Data analysis was performed using the ΔΔCt method. TATA-box binding protein (*Tbp*) was used as the reference gene for normalization. The primer sequences used for qPCR are provided in Supplementary Table 1.

##### Western blots

For AHR protein detection in cell culture experiments, cells were lysed in RIPA buffer (20 mM Tris-HCl [pH 7.5], 150 mM NaCl, 1 mM Na2EDTA, 1 mM EGTA, 1% NP-40, 1% sodium deoxycholate, 2.5 mM sodium pyrophosphate, 1 mM β-glycerophosphate, 1 mM Na_3_VO_4_, 1 µg/ml leupeptin). For detection of hepatic AHR, frozen liver tissue (50–80 mg) was homogenized in 400 µl RIPA buffer. The homogenate was sonicated with Bioruptor on the low setting at 4°C for 5 min, 30 s on, and 30 s off. The samples were then centrifuged at 20 000 × g for 10 min at 4°C. Twenty micrograms of protein were separated by SDS-PAGE and transferred to a PVDF membrane. Membranes were blocked for 2 h at room temperature (RT) in 5% nonfat milk dissolved in Tris-buffered saline (TBS)-0.1% Tween20. Membranes were then incubated overnight at 4°C with anti-AHR (1:10 000; Enzo Life Sciences SA210; lot no. 04011942) followed by incubation with anti-rabbit IgG antibody; 1:6250; 1 h incubation RT. PVDF membranes were stripped and incubated with anti-β-actin antibody 1:4000 (Sigma-Aldrich; A-2228) followed by incubation with anti-mouse IgG antibody for 1 h at RT. After membranes were washed with TBST, SuperSignal West Dura (ThermoScientific) was used for detection. The pixel density of protein bands was analyzed with the ImageJ software (imagej.nih.gov/ij). The corresponding βACTIN band intensity normalized AHR signal.

##### DNA extraction from feces

DNA was extracted from frozen stool samples using QIAamp DNA Stool Mini Kit (QIAGEN, Hilden, Germany) according to the manufacturer’s protocol with the following modifications. Feces were thawed in lysis buffer at 37°C until fully solubilized. To increase the yield of DNA, 600–900 µl of supernatant was passed through the filter column. Differences in the levels of bacteria at the phylum level were determined from fecal DNA by qPCR using PCR primers described previously (Yang *et al.*, 2015). Each reaction consisted of 5 µl KAPA SYBR green, 1 µl DNA with a concentration of 5 ng/μl, 0.1 µl each of forward and reverse primer with a concentration of 10 nM, and 3.8 µl molecular grade H_2_O to a final volume of 10 μl.

#### Statistical analysis

Statistical analysis for significance (*p *<* *0.05) was determined using GraphPad Prism 8.0. Comparison of 2 groups were made with an unpaired, 2-tailed Student’s *t*-test, whereas comparisons of multiple groups were made with analysis of variance (ANOVA) followed by a Tukey’s test. A 2-way ANOVA followed by Tukey’s test was performed when analyzing data with more than one factor.

## Results

###  

#### Mutation of AHR DNA-binding domain prevents TCDD-induced increases of hepatic CYP1A1 and CYP1B1 levels

**Figure 1.**
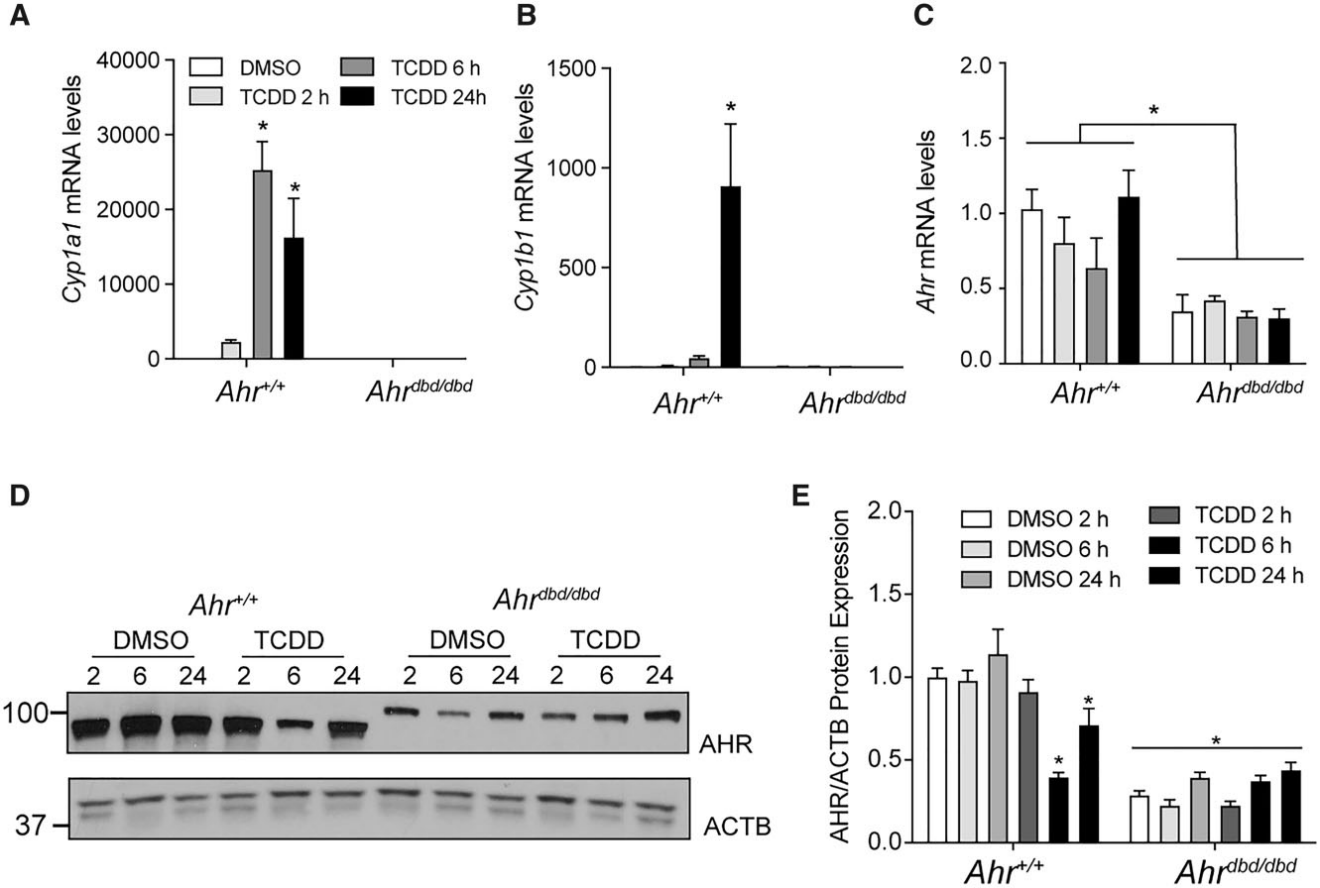
Characterization of hepatic TCDD-induced gene expression in *Ahr^dbd/dbd^* mice and AHR^dbd^ protein levels. Hepatic (A) *Cyp1a1*, (B) *Cyp1b1*, and (C) *Ahr* mRNA levels in *Ahr^+/+^* and *Ahr^dbd/dbd^* after treatment with 100 µg/kg TCDD at the times indicated. D, AHR^wt^ and AHR^dbd^ hepatic protein levels and (E) quantification after treatment with DMSO or 100 µg/kg TCDD at the indicated times. Data represent mean ± SEM (*N* = 3). For A-C, *p ≤ .05 2-way ANOVA compared with DMSO 2 h. For E, *p ≤ .01 2-way ANOVA compared with DMSO 2 h. Abbreviations: DMSO, dimethyl sulfoxide; TCDD, 2,3,7,8-tetrachlorodibenzo-*p*-dioxin.

To confirm that mutation of the AHR DBD prevents TCDD-dependent induction of the *Cyp1a1* and *Cyp1b1 in vivo*, *Ahr^+/+^* and *Ahr^dbd/dbd^* mice were injected intraperitoneally with 100 µg/kg TCDD. After 2, 4, 6, and 24 h, hepatic RNA was isolated and analyzed for *Cyp1a1*, *Cyp1b1*, and *Ahr* expression levels. TCDD-induced *Cyp1a1* and *Cyp1b1* mRNA levels in wild-type mice with peaks at 6 and 24 h, respectively (Figs. 1A and 1B). In contrast, extracts from *Ahr^dbd/dbd^* mice showed very low levels of *Cyp1a1* and *Cyp1b1* that were unaffected by TCDD treatment. *Ahr* mRNA levels were unaltered by TCDD treatment in *Ahr^+/+^* and *Ahr^dbd/dbd^* mice but were significantly lower in *Ahr^dbd/dbd^* than in *Ahr^+/+^* mice (Figure 1C). Western blotting was then used to quantify AHR protein levels in *Ahr^dbd/dbd^* compared with *Ahr^+/+^* mice (Figure 1D). Similar to the mRNA results, AHR protein levels were significantly lower in DMSO-treated *Ahr^dbd/dbd^* mice than in *Ahr*^+/+^ mice at all time points and were not altered after TCDD treatment (Figure 1E). In contrast, TCDD treatment reduced AHR protein expression in wild-type mice at 6 and 24 h, which most likely reflects ligand-induced proteolytic degradation of AHR (Pollenz, 1996). Collectively, these data support previous findings and show that AHR^dbd^ lacks the ability to activate gene transcription from AHRE elements *in vivo* (Bunger *et al.*, 2008). The western blots also revealed that the AHR proteins from *Ahr^+/+^* and *Ahr^dbd/dbd^* were different in size, which is due to the different *Ahr* alleles in the different genotypes.

#### Gene sequencing of AHR transcript from *AHR^wt^* and *AHR^dbd^*

*Ahr^dbd/dbd^* mice were originally generated by mutating the DNA-binding domain of the *Ahr* gene from 129/SvJ embryonic stem cells prior to injection into C57BL/6J blastocysts. Correctly mutated mice were backcrossed for 3 generations to C57BL/6J mice (Bunger *et al.*, 2008), and then for another 10 generations, to C57BL/6J mice (Sarill *et al.*, 2015). Although the *Ahr^dbd/dbd^* mice are congenic C57BL/6J, the *Ahrdbd* gene originated from the 129/SvJ strain that expresses the *Ahr^d^* allele, which is similar to that described for *Ahr^fl/fl^* mice (Wilson *et al.*, 2021). To confirm this, we cloned and sequenced the gene products from *Ahr^+/+^* and *Ahr^dbd/dbd^* mice. The DNA sequences from *Ahr^+/+^* and *Ahr^dbd/dbd^* were aligned with sequences of *Ahr* in C57BL/6J and 129/SvJ that are available on GenBank as AF405563.1 and AF325111.1, respectively. The cloned *Ahr^+/+^* was an exact match with the *Ahr^b1^* from the C57BL/6J strain, whereas the *Ahr^dbd^* was identical with *Ahr^d^* from 129/SvJ, except for the ATC to GGG changes at nucleic acids 73 to 75, and an insertion of GGATCC between nucleic acids 117 and 118. As expected, these 2 artificial mutations in *Ahr^dbd^* result in an I25G substitution as well as a GS insertion after amino acid 39, which are responsible for producing a nonfunctional DNA-binding domain (Bunger *et al.*, 2008). All other differences between the cloned *Ahr^+/+^* and the cloned *Ahr^dbd^* were due to differences between *Ahr^b1^* from C57BL/6J and *Ahr^d^* from 129/SvJ. Compared with the AHR in 129/SvJ mice, the AHR in C57BL/6J mice had: M324I, V375A, P471L, N533S, M589L, and R806X (Supplementary Figure 1). V375A has been shown to be responsible for the enhanced ligand affinity of AHR in C57BL/6, whereas all other changes have no or very limited impact at the functional level (Chang *et al.*, 1993; Poland *et al.*, 1994). The R806X difference results in an AHR protein with only 805 amino acids in C57BL/6J, compared with the 848 amino acids in 129/SvJ.

**Figure 2.**
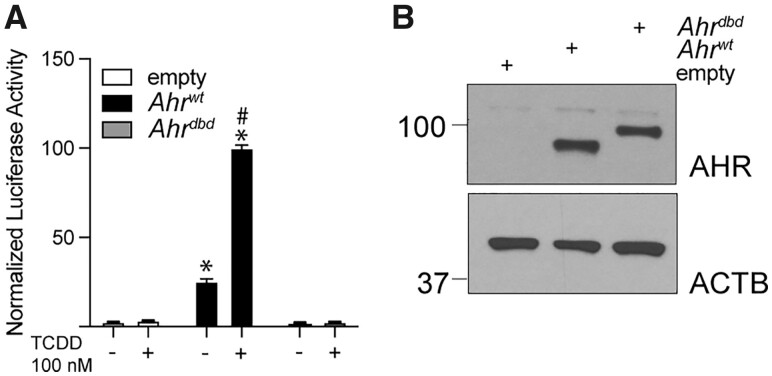
Cloning and characterization of Ahr from *Ahr^+/+^* and *Ahr^dbd/dbd^* mice. A, Relative *Cyp1a1*-regulated luciferase activity of COS-1 cells transfected with empty pcDNA3.1, pcDNA3.1-*Ahr^wt^*, and pcDNA3.1-*Ahr^dbd^* plasmids and treated with DMSO or 100 nM TCDD. B, Western blot of AHR^wt^ and AHR^dbd^ protein after transfection of COS-1 cells. Data represent mean ± SEM (*N* = 3). *p < .05 2-way ANOVA compared with DMSO treated empty plasmid. ^#^*p* < .05 2-way ANOVA compared with transfection matched DMSO treated sample. Abbreviations: DMSO, dimethyl sulfoxide; TCDD, 2,3,7,8-tetrachlorodibenzo-*p*-dioxin.

To determine the ability of AHRs from wild-type and *Ahr^dbd/dbd^* mice to regulate *CYP1A1* reporter gene, COS-1 cells were transfected with pGudLuc 4.1, and pcDNA3.1-*Ahr^wt^*, or pcDNA3.1-*Ahr^dbd^* plasmids. Compared with empty pcDNA3.1, transfection with *Ahr^wt^* alone increased luciferase activity, and this was further increased after TCDD treatment. No significant differences in luciferase activity were observed in the presence of *Ahr^dbd^* transfected cells compared with empty pcDNA3.1. TCDD did not affect luciferase activity in *Ahr^dbd^* transfected cells (Figure 2A). Western blots were used to confirm that the AHR^wt^ and AHR^dbd^ proteins were equally expressed after transfection (Figure 2B). In summary, the AHR^dbd^ has a nonfunctional DNA-binding domain and fails to regulate TCDD-dependent AHR canonical signaling compared with AHR^wt^.

#### Increased sensitivity of *Ahr^dbd/Dbd^* mice to DSS-induced colitis

**Figure 3.**
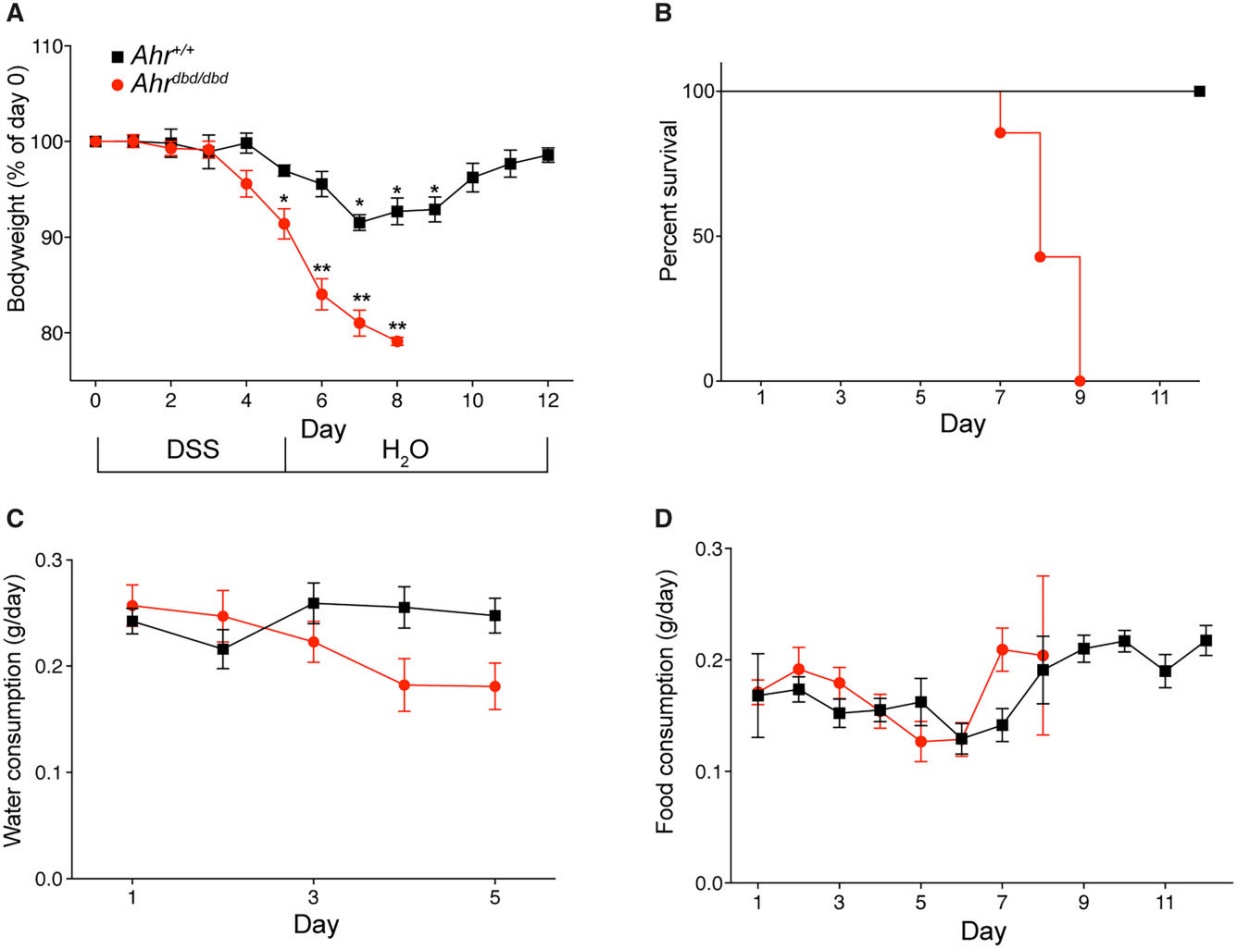
*Ahr^dbd/dbd^* mice have increased sensitivity to DSS-induced colitis. A, Mice were given 2% DSS in the drinking water for 5 days, before switching to water for an additional 7 days. Controls were given water only. Body weight was measured daily. B, Survival curve of DSS-treated mice. (C, Water and (D) food consumption at the days indicated. *p < .01, **p < .001 2-way ANOVA compared with day 1 for each genotype. Abbreviation: DSS, dextran sulfate sodium.

AHR integrates metabolic signals to promote anti-inflammatory responses, intestinal homeostasis, and maintain barrier integrity. Whether the anti-inflammatory actions and overall intestinal protection effects of AHR require its ability to bind to AHREs is not known. To this end, we determined the sensitivity of *Ahr^dbd/dbd^* mice to DSS-induced colitis. Mice were exposed to 2% DSS in their drinking water starting on day 0 and continuing to day 5, at which time the DSS-containing water was replaced with normal water. The mice were then monitored for an additional 7 days until day 12. DSS exposure caused a significant body weight loss in wild-type mice starting on day 7 and continuing through day 9, before recovering by day 10 (Figure 3A). All wild-type animals recovered from the DSS treatment. By contrast, DSS exposure caused a significant body weight loss in *Ahr^dbd/dbd^* mice by day 5 that continued to day 8. None of the DSS-exposed *Ahr^dbd/dbd^* mice survived the treatment as all animals lost greater than 20% of their body weight and were humanely euthanized. No differences in water or food intake between DSS exposed wild-type and *Ahr^dbd/dbd^* mice were observed (Figs. 3B and 3C), suggesting that the body weight loss differences between the genotypes were not due to different exposures to DSS.

**Figure 4.**
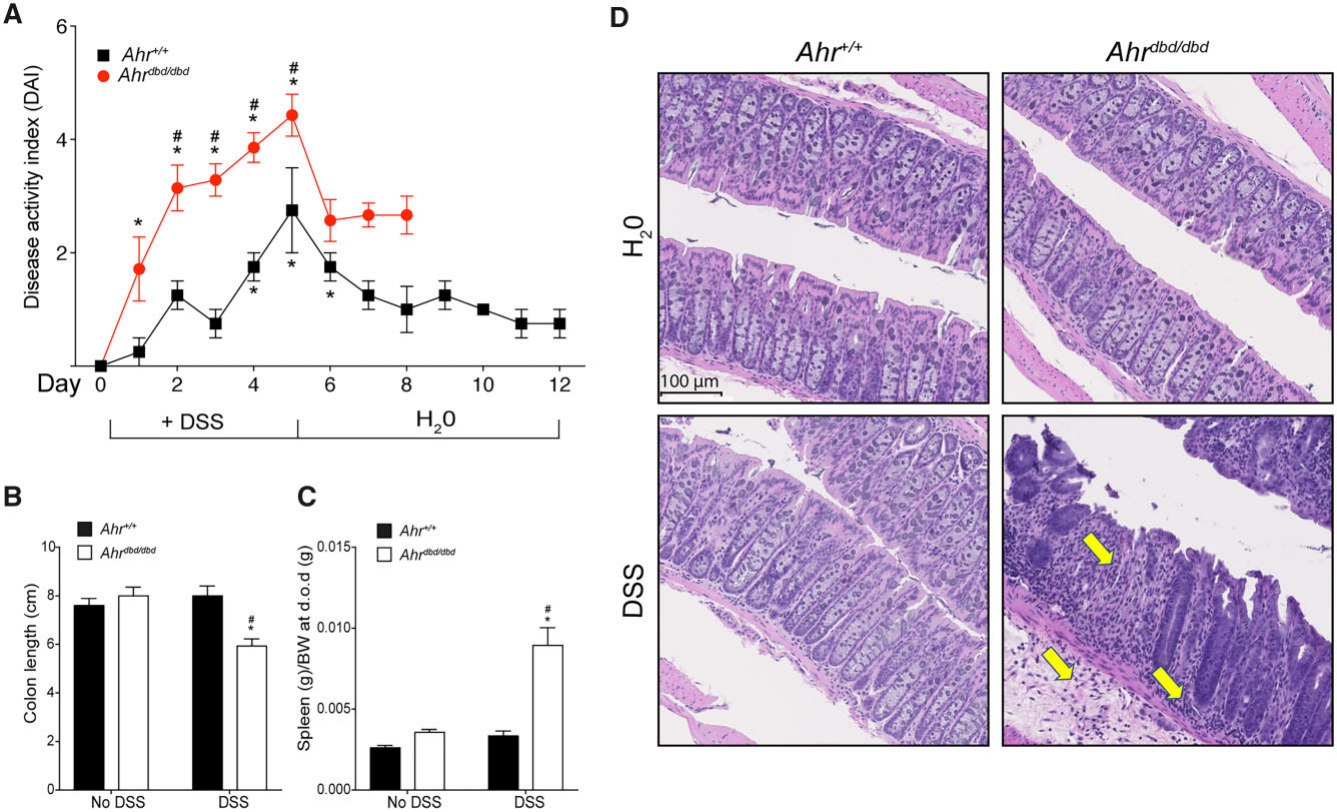
Increased sensitivity of *Ahr^dbd/dbd^* mice to DSS-induced colitis. A, Disease index score (DAI) was assessed daily in all groups. Parameters measured were blood in stool, diarrhea, rectal bleeding, and rectal prolapse. *p <* *.05 2-way ANOVA compared with genotype-matched and no DSS treated mice. ^#^*p *<* *.05 2-way ANOVA compared with day-matched and DSS treated mice. B, Colon length and (C) spleen wet weight corrected for body weight were assessed upon dissection. *p <* *.05 2-way ANOVA compared with genotype-matched and no DSS-treated mice. ^#^*p *<* *.05 2-way ANOVA compared with genotype-matched and DSS-treated mice (D) H&E stain of colon tissue in healthy control mice and mice treated with 2% DSS. *Ahr^dbd/dbd^* mice exhibit extensive tissue damage and immune cell infiltration compared with *Ahr^+/+^* mice. The arrows indicate infiltration of inflammatory cells and crypt loss. Abbreviation: DSS, dextran sulfate sodium.

We next assessed the severity of inflammation using a daily disease activity index (DAI) assessment (Figure 4A) as described previously (Hutin *et al.*, 2022; Kim *et al.*, 2012). DSS exposed *Ahr^dbd/dbd^* mice displayed a significantly greater DAI than wild-type mice. Colon lengths were reduced in *Ahr^dbd/dbd^* mice but not in wild-type mice after DSS exposure (Figure 4B). DSS-exposed *Ahr^dbd/dbd^* mice had increased spleen weight compared with DSS exposed *Ahr^+/+^* mice and *Ahr^dbd/dbd^* mice given water only (Figure 4C). Histological analysis of colon sections from *Ahr^dbd/dbd^* mice exposed to DSS for 7–9 days displayed prototypical features of colitis, including regions of major crypt loss and mucosal inflammation with neutrophilic infiltration (Figure 4D). In contrast, day 12 *Ahr^+/+^* mice displayed normal colon mucosal morphology with little evidence for crypt damage and immune cell infiltration. Vehicle-treated mice from both genotypes displayed normal colon mucosal morphology.

#### Increased expression of proinflammatory genes in DSS-treated *Ahr^dbd/Dbd^* compared with *Ahr^+/+^* mice

**Figure 5.**
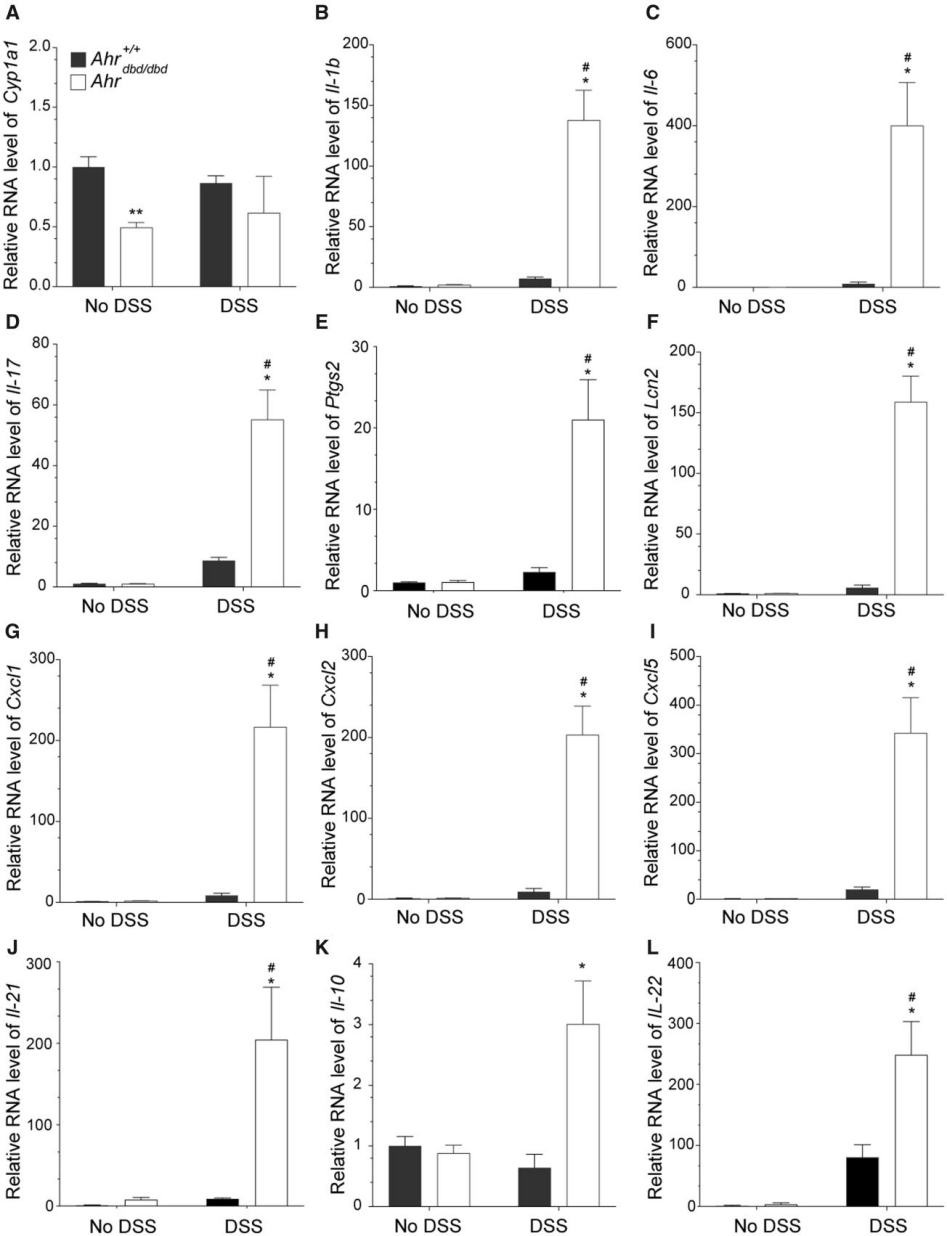
Increased expression of proinflammatory genes in DSS-exposed *Ahr^dbd/dbd^* compared with wild-type mice. Intestinal mRNA expression levels of (A) *Cyp1a1*, (B) *Il-1b*, (C) *Il-6*, (D) *Il-17*, (E) *Ptgs2*, (F) *Lcn2* (G) *Cxcl1*, (H) *Cxcl2*, (I) *Cxcl5*, (J) *Il-21*, (K) *Il-10*, and (L) *Il-22* in colon tissue isolated from *Ahr^+/+^* and *Ahr^dbd/dbd^* mice that were not exposed to DSS (No DSS) or exposed to 2% DSS in their drinking water. The relative mRNA levels of the indicated genes were determined with RT-qPCR. **p < .05 Student’s *t*-test compared with genotype and treatment-matched *Ahr^+/+^* mice. *p < .05 2-way ANOVA compared with genotype-matched and DSS-treated animals. ^#^*p* < .05 2-way ANOVA compared with treatment-matched *Ahr^+/+^* mice. Abbreviation: DSS, dextran sulfate sodium.

We then examined the mRNA levels of *Cyp1a1*, several cytokines, chemokines, prostaglandin synthase 2 (*Ptgs2*), and lipocalin-2 (*Lcn2*), a potential biomarker for IBD. *Cyp1a1* mRNA levels in water control *Ahr^dbd/dbd^* mice were lower than wild-type mice, but this difference was lost after DSS treatment (Figure 5A). The levels of all cytokines and chemokines examined, as well as *Ptgs2* and *Lcn2*, were significantly increased in colon tissue from DSS-treated *Ahr^dbd/dbd^* compared with wild-type mice (Figs. 5B–L). These data demonstrate that the increased sensitivity to DSS-induced colitis in mice expressing a DNA-binding deficient AHR is due to hyperinflammation.

#### Microbial composition is similar in *Ahr^+/+^* and *Ahr^dbd/Dbd^* mice

**Figure 6.**
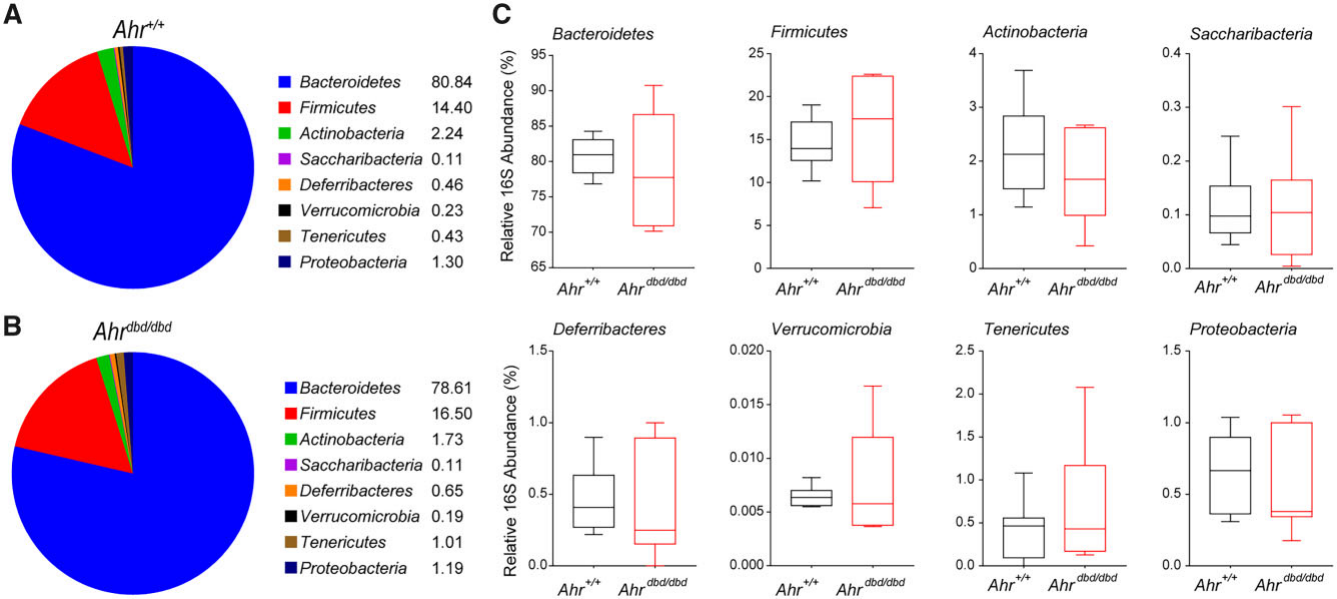
Microbiota at the phylum level were measured before the start of experiment to assess differences between genotypes. A, Average relative amounts of bacterial phyla in healthy *Ahr^+/+^* mice. B, Average relative amounts of bacterial phyla in healthy *Ahr^dbd/dbd^* mice. Detailed view of average amounts in (C) *Bacteroidetes*, *Firmicutes*, *Actinobacteria*, *Saccharibacteria*, *Deferribacteres*, *Verrucomicrobia*, *Tenericutes*, and *Proteobacteria*.

We next analyzed the microbial composition at the phylum level using a qPCR-based approach to determine if the *Ahr^dbd/dbd^* mice exhibit a dysbiosis that predisposes them to intestinal inflammation. For these studies, we analyzed feces from water control animals for both genotypes (Figs. 6A and 6B). We did not observe any significant differences in microbial composition between genotypes (Figure 6C).

## Discussion

AHR is a key regulator of intestinal homeostasis and barrier function by promoting an anti-inflammatory intestinal environment (Busbee *et al.*, 2013; Cho and Kelsall, 2014; Stockinger *et al.*, 2014). Loss of AHR expression or reduced endogenous AHR ligand levels in mice increases their susceptibility to DSS-induced colitis or *Citrobacter rodentium* infection by dampening inflammatory responses (Stockinger *et al.*, 2021). However, whether canonical AHR signaling and AHR-AHRE binding is required for these outcomes is not known. We show here that mice harboring a DNA-binding deficient AHR, which is incapable of binding to AHREs, exhibit a similar sensitivity to DSS-induced colitis as *Ahr^−/−^* mice. Our findings reveal the importance of canonical AHR signaling, and specifically AHR-AHRE binding in protecting against intestinal inflammation and DSS-induced colitis.

Canonical AHR signaling is fundamental for the biological and toxicological effects of AHR ligands (Bunger *et al.*, 2003, 2008), including immune regulation as many cytokines have AHREs in their upstream regulatory regions (DiNatale *et al.*, 2010). Interactions between AHR and musculoaponeurotic fibrosarcoma (cMAF) are important for the regulation of *Il-10* and *Il-21* in the development of T cell subset, Tr1 cells (Apetoh *et al.*, 2010). Moreover, lipopolysaccharide-induced increases in *Il-10* are impaired in macrophages isolated from *Ahr^−/−^* mice (Zhu *et al.*, 2018). IL-10 protects against intestinal inflammation by preventing pro-inflammatory responses and maintaining mucosal homeostasis. This is supported by the findings that *Il-10^−/−^* and IL-10 receptor b null (*Ilbrb^−/−^*) mice develop spontaneous enterocolitis (Kuhn *et al.*, 1993; Spencer *et al.*, 1998), and that polymorphisms in *IL-10R* and *IL-10* are associated with UC in early childhood (Engelhardt and Grimbacher, 2014). IL-21 is produced by T cells and natural killer T cells and effects a wide range of immune and nonimmune cells. Treatment with DSS increases intestinal IL-21 levels, whereas DSS-treated *Il-21^ko^* mice have reduced gut pathology, lower immune cell infiltration, and reduced IL-17 production compared with wild-type mice (Fina *et al.*, 2008). Neutralization of IL-21 ameliorates clinical and pathological findings primarily in experimental T cell-driven colitis. However, IL-21 may play a protective role in the DSS-induced colitis model through its induction of IL-22, a member of the IL-10 family that promotes tissue regeneration and repair (Wei *et al.*, 2020). Thus, it is possible that the increased *Il-10* and *Il-21* mRNA levels observed in DSS-exposed *Ahr^dbd/dbd^* compared *Ahr^+/+^* mice may be due to the increased inflammation in the DNA-binding deficient AHR mice, and not necessarily due to AHRE-independent regulation of these cytokines by AHR.

AHR plays an essential role in the development and maintenance of intestinal RORγt^+^ type 3 innate lymphoid cells (ILC3s), which are important for gut immunity through their production of IL-22. IL-22 plays a crucial role in the early phase of host defense against *C. rodentium* infection and barrier integrity (Dumoutier *et al.*, 2000; Sugimoto *et al.*, 2008; Zheng *et al.*, 2008). DSS exposed *Il-22^−/−^* mice exhibit increased inflammation, more severe weight loss, and impaired recovery compared with wild-type mice (Zindl *et al.*, 2013). Moreover, exposure of *Ahr^−/−^* or CYP1A1 transgenic mice (endogenous AHR ligand deficient) to *C. rodentium* results in reduced barrier integrity and lower IL-22 levels, increased inflammation and mortality (Qiu *et al.*, 2012; Schiering *et al.*, 2017). AHR-dependent increases in IL-22 levels are also lost in colon explant cultures from *Ahr^−/−^* or CYP1A1 transgenic mice (Schiering *et al.*, 2017), whereas CD4^+^ T cells isolated from *Ahr^b-1^* mice have higher IL-21-dependent increases in IL-22 compared with *Ahr^d^* mice (Yeste *et al.*, 2014). Taken together, these studies support a role for AHR in the regulation of IL-22. Although we did not determine the number of intestinal ILC3s in *Ahr^dbd/dbd^* mice, we observed that *Il-22* levels were increased in DSS-exposed *Ahr^dbd/dbd^* compared with *Ahr^+/+^* mice. This suggests that AHR might regulate Il-22 in an AHRE-independent manner. Many different types of immune cells also produce IL-22 including αβ and γδ T cells, and NKT cells (Parks *et al.*, 2015; Sonnenberg *et al.*, 2011a,b). However, in models of colitis and skin inflammation several cytokines and chemokines are increased in *Ahr^−/−^* compared with *Ahr^+/+^* mice (Di Meglio *et al.*, 2014; Wang *et al.*, 2018). Thus, we cannot exclude that the increased *Il-22* levels observed in DSS-exposed *Ahr^dbd/dbd^* mice might be due to other immune cells independently of AHR, as has been reported in skin inflammation models (Di Meglio *et al.*, 2014).

*Ahr^dbd/dbd^* mice express the *Ahr^d^* allele with a I25G mutation and GS insertion after amino acid 39 which abolishes AHRE binding (Bunger *et al.*, 2008). C57BL/6 mice harboring *Ahr^d^* allele fed purified rodent chow (AIN-93G) and exposed to 3.5% DSS were reported to exhibit similar weight loss and DAI compared with correspondingly fed and DSS exposed C57BL/6 *Ahr^b-1^* mice (Hubbard *et al.*, 2017). DSS-induced weight loss was delayed, and DAI reduced in C57BL/6 *Ahr^b-1^* mice fed a diet rich in AHR ligands (broccoli diet). C57BL/6 *Ahr^d^* broccoli-fed mice exhibit some improvement in weight loss but no change in DAI, suggesting that AHR ligand rich diets are not as effective at protecting against DSS-induced colitis in mice that harbor the *Ahr^d^* allele. In another study, exposure of C57BL/6 *Ahr^+/+^* (*Ahr^b-1^*) to 3% DSS resulted in rapid weight loss in standard chow fed mice followed by full recovery after DSS withdrawal (Li *et al.*, 2011). In contrast, similarly exposed *Ahr^−/−^* mice experienced accelerated weight loss and extreme colon shortening with 13 out of 16 mice losing over 20% of body weight by 1 day after DSS withdrawal (Li *et al.*, 2011). The sensitivity of *Ahr^dbd/dbd^* mice to DSS-induced colitis we observed in the present study is very similar with that reported for *Ahr^−/−^* mice fed standard chow (Li *et al.*, 2011). Taken together, this suggests that the increased sensitivity of *Ahr^dbd/dbd^* mice to DSS-induced colitis is due to the expression of DNA-binding deficient AHR rather than reduced ligand affinity of AHR^dbd^ compared with AHR^wt^ protein.

Genome-wide profiling of AHR-binding sites revealed that the majority of AHR bound regions do not contain AHREs (Dere *et al.*, 2011; Lo and Matthews, 2012). AHR regulates several cellular pathways through interactions with important regulatory proteins and transcription factors. AHR interacts with retinoblastoma protein (pRB) resulting in G1 cell-cycle arrest (Puga *et al.*, 2002). Ligand-activated AHR also regulates inflammation through nongenomic pathways. TCDD- and polycyclic aromatic hydrocarbon (PAH)-treatment results in activation of MAPK, and Ca^2+^-induced cPLA2 and PTGS2 activation generating inflammatory prostaglandins (Puga *et al.*, 1997). AHR modulates SRC-dependent STAT3 phosphorylation and the subsequent production of the anti-inflammatory cytokine IL-10 (Zhu *et al.*, 2018). In addition, AHR inhibits the PI3K-AKT signaling by increasing the ubiquitination of RAC1 which results in a mitigated inflammatory response in macrophages (Grosskopf *et al.*, 2021). Although there are no changes in the nuclear localization sequence or nuclear export sequence between AHR^wt^ and AHR^dbd^, the AHR^dbd^ protein has been reported to be located exclusively in the nucleus (Bunger *et al.*, 2008). Therefore, in addition to the inability of AHR to bind to AHREs, cytosolic AHR signaling is also impaired in *Ahr^dbd/dbd^* mice. We cannot exclude that cytosolic AHR signaling may contribute to the anti-inflammatory and protective role of AHR in the gut. To test this, we could perform similar DSS exposure studies in *Ahr^nls/nls^* mice that harbor a mutation in the nuclear localization sequence of AHR (Bunger *et al.*, 2003). These mice are resistant to TCDD-induced toxicity and show similar development abnormalities as *Ahr^−/−^* mice, but their barrier integrity and sensitivity to colitis have not been studied. Like *Ahr^dbd/dbd^* mice, *Ahr^nls/nls^* mice were generated using embryonic stem cells derived from 129Sv mice and harbor the *Ahr^d^* allele. The original *Ahr^+/nls^* mice were backcrossed onto C57BL/6-*Ahr^d^* mice to generate appropriate controls (Bunger *et al.*, 2003). This strategy prevented the potential confounding results that may arise when comparing mice expressing *Ahr^b-1^* with *Ahr^nls/nls^ (Ahr^d^)*. A similar backcrossing strategy onto C57BL/6-*Ahr^d^* mice could be done for the *Ahr^dbd/dbd^* mice. Alternatively, target gene editing strategies could be used to convert the *Ahr^dbd/dbd^* (*Ahr^d^*) to *Ahr^dbd/dbd^* (*Ahr^b-1^*) as have been described for *Ahr* conditional null (*Ahr^fx^*) mice (Wilson *et al.*, 2021). Regardless, both approaches would provide appropriate *Ahr* allele control animals and eliminate one of the weaknesses of using the congenic C57BL/6 *Ahr^dbd/dbd^* strain in the present study.

Dysbiosis to a less diverse and less beneficial composition is commonly observed in IBD. Patients with IBD present with increased *Proteobacteria*, whereas *Firmicutes* are often reduced in amount and diversity (Frank *et al.*, 2007). AHR expression and its activation help shape the composition of the microbiota, whereas several microbial produced metabolites act as AHR ligands or activators (Stockinger *et al.*, 2021). In contrast to reports of dysbiosis between mice expressing *Ahr^d^* compared with those expressing *Ahr^b-1^* and between *Ahr^−/−^* and *Ahr^+/+^* mice (Hubbard *et al.*, 2017; Yang *et al.*, 2020), we did not observe any significant differences in microbiota composition at the phylum level between *Ahr^dbd/dbd^* and *Ahr^+/+^* mice. It is possible that if metagenomic analyses of the microbiomes were done, we might have detected differences in microbial communities between *Ahr^dbd/dbd^* and *Ahr^+/+^* mice.

In summary, our findings show that AHR-AHRE interactions are required to reduce inflammation, maintain a healthy intestinal environment, and protect against DSS-induced colitis. These findings further support targeting AHR for the therapeutic treatment of inflammatory diseases, including IBD.

## Supplementary Material

kfac132_Supplementary_Data
