# Supplementary material for: Increased sensitivity to chemically induced colitis in mice harboring a DNA-binding deficient aryl hydrocarbon receptor
Source: Toxicol Sci. 2022 Dec 15;191(2):321–31. doi: 10.1093/toxsci/kfac132 (PMC9936212; doi:10.1093/toxsci/kfac132)
Supplement: kfac132_Supplementary_Data [file kfac132_supplementary_data.zip › kfac132_Supplementary_Data/toxsci-22-0351-File008.pdf]

Supplementary Table S1. RT-qPCR primers used to determine changes in expression levels in colon tissue from wild-type of $Ahr^{dbd/dbd}$ mice +/- DSS exposure.

	Forward	Reverse
Cyp1a1	CGTTATGACCATGATGACCAAGA	TCCCCAAACTCATTGCTCAGAT
Il-1b	GGACCCATATGAGCTGAAAGCT	TGTCGTTGCTTGGTTCTCCTT
Il-6	TGATGCACTTGCAGAAAACA	ACCAGAGGAAATTTTCAATAGGC
Il-17	CTCTGTGATCTGGGAAGCTCAGT	CTTTCCCTCCGCATTGACA
Ptgs2	TGTGAACAATCAAACAAAATGATG	GCGTAAATTCC ACAGCCTAAGT
Lcn2	AAGGCAGCTTTACGATGTACAGC	CTTGCACATTGTAGCTGTGTACC
Cxcl1	CCGAAGTCATAGCCACACTCA	CTCCGTTACTTGGGGACACC
Cxcl2	TGAACAAAGGCAAGGCTAACTG	CAGGTACGATCCAGGCTTCC
Cxcl5	GCCCCTTCCTCAGTCATAGC	AGCTTTCTTTTTGTCACTGCCC
Il-21	GCCTCCTGATTAGACTTCGTCAC	CAGGCAAAAGCTGCATGCTCAC
Il-10	AACTGCACCCACTTCCCAGTC	CATTAAGGAGTCGGTTAGCAG
Il-22	GAGGCCAGCCTTGCAGATAA	CCTTAGCACTGACTCCTCGG
