# Supplementary material for: Increased sensitivity to chemically induced colitis in mice harboring a DNA-binding deficient aryl hydrocarbon receptor
Source: Toxicol Sci. 2022 Dec 15;191(2):321–31. doi: 10.1093/toxsci/kfac132 (PMC9936212; doi:10.1093/toxsci/kfac132)
Supplement: kfac132_Supplementary_Data [file kfac132_supplementary_data.zip › kfac132_Supplementary_Data/toxsci-22-0351-File009.pdf]

```
C57BL_6J     1 MSSGANITYASRKRRKPVQKTVKPIPAEGIKSNPSKRHR--DRLNTELDRLASLLPFPQDVINKLDKLSV
Ahr_wt       1 MSSGANITYASRKRRKPVQKTVKPIPAEGIKSNPSKRHR--DRLNTELDRLASLLPFPQDVINKLDKLSV
129_SvJ      1 MSSGANITYASRKRRKPVQKTVKPIPAEGIKSNPSKRHR--DRLNTELDRLASLLPFPQDVINKLDKLSV
Ahr_dbd      1 MSSGANITYASRKRRKPVQKTVKPGPAEGIKSNPSKRHRGSDRLNTELDRLASLLPFPQDVINKLDKLSV

C57BL_6J    69 LRLSVSYLRAKSFFDVALKSTPADRNGGQDQCRAQIRDWQDLQEGEFLLQALNGFVLVVTADALVFYASS
Ahr_wt      69 LRLSVSYLRAKSFFDVALKSTPADRNGGQDQCRAQIRDWQDLQEGEFLLQALNGFVLVVTADALVFYASS
129_SvJ     69 LRLSVSYLRAKSFFDVALKSTPADRNGGQDQCRAQIRDWQDLQEGEFLLQALNGFVLVVTADALVFYASS
Ahr_dbd     71 LRLSVSYLRAKSFFDVALKSTPADRNGGQDQCRAQIRDWQDLQEGEFLLQALNGFVLVVTADALVFYASS

C57BL_6J   139 TIQDYLGFQQSDVIHQSVYELIHTEDRAEFQRQLHWALNPDSAQGVDEAHGPPQAAVYYTPDQLPPENAS
Ahr_wt     139 TIQDYLGFQQSDVIHQSVYELIHTEDRAEFQRQLHWALNPDSAQGVDEAHGPPQAAVYYTPDQLPPENAS
129_SvJ    139 TIQDYLGFQQSDVIHQSVYELIHTEDRAEFQRQLHWALNPDSAQGVDEAHGPPQAAVYYTPDQLPPENAS
Ahr_dbd    141 TIQDYLGFQQSDVIHQSVYELIHTEDRAEFQRQLHWALNPDSAQGVDEAHGPPQAAVYYTPDQLPPENAS

C57BL_6J   209 FMERCFRCRLRCLLDNSSGFLAMNFQGRLKYLHGQNKKGKDGALLPPQLALFAIATPLQPPSILEIRTKN
Ahr_wt     209 FMERCFRCRLRCLLDNSSGFLAMNFQGRLKYLHGQNKKGKDGALLPPQLALFAIATPLQPPSILEIRTKN
129_SvJ    209 FMERCFRCRLRCLLDNSSGFLAMNFQGRLKYLHGQNKKGKDGALLPPQLALFAIATPLQPPSILEIRTKN
Ahr_dbd    211 FMERCFRCRLRCLLDNSSGFLAMNFQGRLKYLHGQNKKGKDGALLPPQLALFAIATPLQPPSILEIRTKN

C57BL_6J   279 FIFRTKHKLDFTPIGCDAKGQLILGYTEVELCTRGSGYQFIHAADILHCAESHIRMIKTGESGMTVFRLL
Ahr_wt     279 FIFRTKHKLDFTPIGCDAKGQLILGYTEVELCTRGSGYQFIHAADILHCAESHIRMIKTGESGMTVFRLL
129_SvJ    279 FIFRTKHKLDFTPIGCDAKGQLILGYTEVELCTRGSGYQFIHAADMLHCAESHIRMIKTGESGMTVFRLL
Ahr_dbd    281 FIFRTKHKLDFTPIGCDAKGQLILGYTEVELCTRGSGYQFIHAADMLHCAESHIRMIKTGESGMTVFRLL

C57BL_6J   349 AKHSRWRWVQSNARLIYRNGRPDYIIATQRPLTDEEGREHLQKRSTSLPFMFATGEAVLYEISSPFSPIM
Ahr_wt     349 AKHSRWRWVQSNARLIYRNGRPDYIIATQRPLTDEEGREHLQKRSTSLPFMFATGEAVLYEISSPFSPIM
129_SvJ    349 AKHSRWRWVQSNARLIYRNGRPDYIIVTQRPLTDEEGREHLQKRSTSLPFMFATGEAVLYEISSPFSPIM
Ahr_dbd    351 AKHSRWRWVQSNARLIYRNGRPDYIIVTQRPLTDEEGREHLQKRSTSLPFMFATGEAVLYEISSPFSPIM

C57BL_6J   419 DPLPIRTKSNTSRKDWAPQSTPSKDSFHPSSLMSALIQQDESIYLCPPSSPALLDSHFLMGSVSKCGSWQ
Ahr_wt     419 DPLPIRTKSNTSRKDWAPQSTPSKDSFHPSSLMSALIQQDESIYLCPPSSPALLDSHFLMGSVSKCGSWQ
129_SvJ    419 DPLPIRTKSNTSRKDWAPQSTPSKDSFHPSSLMSALIQQDESIYLCPPSSPAPLDSHFLMGSVSKCGSWQ
Ahr_dbd    421 DPLPIRTKSNTSRKDWAPQSTPSKDSFHPSSLMSALIQQDESIYLCPPSSPAPLDSHFLMGSVSKCGSWQ

C57BL_6J   489 DSFAAAGSEAALKHEQIGHAQDVNLALSGGPSELFPDNKNNDLYSIMRNLGIDFEDIRSMQNEEFFRTDS
Ahr_wt     489 DSFAAAGSEAALKHEQIGHAQDVNLALSGGPSELFPDNKNNDLYSIMRNLGIDFEDIRSMQNEEFFRTDS
129_SvJ    489 DSFAAAGSEAALKHEQIGHAQDVNLALSGGPSELFPDNKNNDLYNIMRNLGIDFEDIRSMQNEEFFRTDS
Ahr_dbd    491 DSFAAAGSEAALKHEQIGHAQDVNLALSGGPSELFPDNKNNDLYNIMRNLGIDFEDIRSMQNEEFFRTDS

C57BL_6J   559 TAAGEVDFKDIDITDEILTYVQDSLNNSTLLNSACQQQPVTQHLSCMLQERLQLEQQQQLQQPPPQALEP
Ahr_wt     559 TAAGEVDFKDIDITDEILTYVQDSLNNSTLLNSACQQQPVTQHLSCMLQERLQLEQQQQLQQPPPQALEP
129_SvJ    559 TAAGEVDFKDIDITDEILTYVQDSLNNSTLMNSACQQQPVTQHLSCMLQERLQLEQQQQLQQPPPQALEP
Ahr_dbd    561 TAAGEVDFKDIDITDEILTYVQDSLNNSTLMNSACQQQPVTQHLSCMLQERLQLEQQQQLQQPPPQALEP

C57BL_6J   629 QQQLCQMVCPQQDLGPKHTQINGTFASWNPTPPVSFNCPQQELKHYQLFSSLQGTAQEFPYKPEVDSVPY
Ahr_wt     629 QQQLCQMVCPQQDLGPKHTQINGTFASWNPTPPVSFNCPQQELKHYQLFSSLQGTAQEFPYKPEVDSVPY
129_SvJ    629 QQQLCQMVCPQQDLGPKHTQINGTFASWNPTPPVSFNCPQQELKHYQLFSSLQGTAQEFPYKPEVDSVPY
Ahr_dbd    631 QQQLCQMVCPQQDLGPKHTQINGTFASWNPTPPVSFNCPQQELKHYQLFSSLQGTAQEFPYKPEVDSVPY

C57BL_6J   699 TQNFAPCNQPLLPEHSKSVQLDFPGRDFEPSLHPTTSNLDFVSCLQVPENQSHGINSQSAMVSPQAYYAG
Ahr_wt     699 TQNFAPCNQPLLPEHSKSVQLDFPGRDFEPSLHPTTSNLDFVSCLQVPENQSHGINSQSAMVSPQAYYAG
129_SvJ    699 TQNFAPCNQPLLPEHSKSVQLDFPGRDFEPSLHPTTSNLDFVSCLQVPENQSHGINSQSAMVSPQAYYAG
Ahr_dbd    701 TQNFAPCNQPLLPEHSKSVQLDFPGRDFEPSLHPTTSNLDFVSCLQVPENQSHGINSQSAMVSPQAYYAG

C57BL_6J   769 AMSMYQCQPGPQRTPVDQTQYSSEIPGSQAFLSKVQS---------------------------------
Ahr_wt     769 AMSMYQCQPGPQRTPVDQTQYSSEIPGSQAFLSKVQS---------------------------------
129_SvJ    769 AMSMYQCQPGPQRTPVDQTQYSSEIPGSQAFLSKVQSRGIFNETYSSDLSSIGHAAQTTGHLHHLAEARP
Ahr_dbd    771 AMSMYQCQPGPQRTPVDQTQYSSEIPGSQAFLSKVQSRGIFNETYSSDLSSIGHAAQTTGHLHHLAEARP

C57BL_6J       ----------
Ahr_wt         ----------
129_SvJ    839 LPDITPGGFL
Ahr_dbd    841 LPDITPGGFL
```

Supplementary Figure S1 Amino acid sequence alignment of the cloned AHR^{wt} and AHR^{dbd} proteins. AHR sequences in the C57BL/6J strain (*Ahr^{b1}*) and the 129/SvJ strain (*Ahr^{d}*) are available at GenBank as AAL89728.1 and AAK13443.1, respectively. Conserved residues are in black, conservative changes are in grey, and non-conservative mutations are in white.
